# deepBlink: threshold-independent detection and localization of diffraction-limited spots

**DOI:** 10.1093/nar/gkab546

**Published:** 2021-07-01

**Authors:** Bastian Th Eichenberger, YinXiu Zhan, Markus Rempfler, Luca Giorgetti, Jeffrey A Chao

**Affiliations:** Friedrich Miescher Institute for Biomedical Research, 4058 Basel, Switzerland; University of Basel, 4003 Basel, Switzerland; Friedrich Miescher Institute for Biomedical Research, 4058 Basel, Switzerland; Friedrich Miescher Institute for Biomedical Research, 4058 Basel, Switzerland; Friedrich Miescher Institute for Biomedical Research, 4058 Basel, Switzerland; Friedrich Miescher Institute for Biomedical Research, 4058 Basel, Switzerland

## Abstract

Detection of diffraction-limited spots in single-molecule microscopy images is traditionally performed with mathematical operators designed for idealized spots. This process requires manual tuning of parameters that is time-consuming and not always reliable. We have developed deepBlink, a neural network-based method to detect and localize spots automatically. We demonstrate that deepBlink outperforms other state-of-the-art methods across six publicly available datasets containing synthetic and experimental data.

## INTRODUCTION

In biomedical research, the detection, counting and localization of sub-diffraction fluorescent signals (spots) represent essential steps in various imaging applications including particle tracking in live cell imaging data and quantification of mRNAs in single-molecule fluorescent *in situ* hybridization (smFISH). While advances in smFISH-based spatial transcriptomics have enabled the quantification of thousands of mRNAs in single cells, these methodologies have focused on the development of transcript barcoding strategies for multiplexing and have relied on conventional threshold-based detection of single mRNA molecules (1,2). Accurate, high-throughput spot detection and localization in single-molecule fluorescent microscopy images with varying background brightness levels and spot qualities, however, poses a challenge for current spot detection methods. Current methods such as the broadly adopted TrackMate (3) are based on intensity thresholds and rely on mathematical operators (e.g. Laplacian of Gaussian) that require ad-hoc adjustments of parameters on a cell-by-cell and image-by-image basis. These adjustments are time consuming and not always reproducible as they rely on subjective selection of thresholds. Fully automated, user-friendly, accurate, and reproducible spot detection and localization methods are currently not available.

Deep Learning is a machine learning method that uses artificial neural networks to automatically learn features and patterns from raw data. Even though deep learning requires labeled data, once a model is trained, it can be used out of the box without further modifications or parameter adjustments. Recent advances in deep learning have consolidated the convolutional neural network (CNN) as the state-of-the-art for computer vision applications (4,5). Previously, CNNs have been used for threshold-independent particle detection (6–8), however, to the extent of our knowledge, none of these approaches can localize particle positions with sub-pixel resolution. This prevents accurate positional measurements and thus their application in high-resolution microscopy. In addition, while the source code of some methods is available, none of them have been made easily accessible for non-experts.

Here we present deepBlink, a command-line interface to automatically detect and localize diffraction-limited spots. deepBlink exploits neural networks to achieve sub-pixel localization of spots in microscopy images. By benchmarking deepBlink against state-of-the-art methods in spot detection across multiple datasets with different signal-to-noise ratios (SNRs), we show that deepBlink can detect spots more accurately and with higher precision.

## MATERIALS AND METHODS

### Training data

Datasets ‘Microtubule’, ‘Receptor’, and ‘Vesicle’ were created from the ISBI Particle Tracking Challenge 2012 (9). Each dataset consisted of 1200 images and was split into training/validation/test splits ensuring equal distribution of signal to noise ratios and densities. The synthetic ‘Particle’ dataset was generated using the ‘Synthetic Data Generator’ Fiji plugin (http://smal.ws/wp/software/synthetic-data-generator/) described in Smal *et al.* (10). A pool of 576 images was created with various signal to noise ratios and background intensities to mimic small diffraction-limited particles. The ‘smFISH’ dataset was created from a pool of 643 manually annotated smFISH images originally published in Horvathova *et al.* (11). The ‘SunTag’ dataset consists of 544 manually annotated live cell SunTag images originally published in Mateju *et al.* (12).

### Dataset labeling

Labeling for both the experimental smFISH and SunTag datasets was performed using TrackMate. Each image was subjected to manual thresholding and falsely detected spots were manually corrected. Labels were exported using the ‘All Spots statistics’ tool. Once all images were labeled, the deepBlink command deepBlink create was used to convert all images and labels into a partitioned dataset file.

### Network architecture

deepBlink’s default network is based on the U-Net architecture (13). We used a second encoder to reduce the fully sized representation into a smaller one corresponding to the cell-size. This second encoder is variable in its depth to adjust for the configured cell-size. Each encoding step consists of a convolutional block, squeeze and excitation block, and spatial dropout (Supplementary Figure S1 and Supplementary Table S1). Decoding steps had the same layout but did not employ dropout. The architecture was implemented in Python version 3.7 using the TensorFlow framework version 2.2.

### Network training

All deepBlink trainings were performed using the public command-line interface on version 0.1.0 on the default settings provided by deepBlink config. In particular, we used a cell-size of 4, a dropout rate of 0.3, a batch size of 2, a learning rate of 0.0001, and the AMSGrad stochastic optimizer (14). Training was run on a computer cluster with the following specifications: 64-bit CentOS Linux 7 (Core), 96 cores (3926 threads) Intel(R) Xeon(R) Platinum 8168 CPU 2.70 GHz, 1007 GB RAM, 8 Nvidia GeForce RTX 2080 Ti GPUs (11 GB VRAM). Training on average took around 5 h per model. All models were trained for 200 epochs.

### Benchmarking of TrackMate

TrackMate (3) is not a machine learning method and relies on manual thresholding. To find the best parameters, we optimized TrackMate on the pool of training and validation images from each dataset. More specifically, we performed a grid search across different values of ‘Estimated blob diameters’ in the ‘LoG’ filter option in Fiji. Subsequently, the resulting quality scores were normalized to the median of all images. Twenty linearly distributed quantiles were applied. At each quantile, the F1 integral score was calculated for each image. The finally selected parameters (blob diameter and absolute quantile value) maximized the mean F1 score on the combined training and validation dataset.

### Benchmarking of DetNet

Due to the lack of a publicly available implementation of DetNet (8), we implemented the model and followed the training procedure in the publication. Notably, the number of parameters in our model was 10x larger than originally described. Using the coordinate locations, ground truth output masks were created with a single pixel of value one at the centroid position. The output segmentation maps were converted back to coordinates by binarizing and taking the centroids of each fully connected component. A hyperparameter search was performed across twenty linearly distributed sigmoid shifts α. The α with the highest validation F1 score was used for evaluation on the test set. Supplementary Table S6 lists the α values used for each dataset.

### Benchmarking of deepBlink

deepBlink was trained through the command-line interface version 0.1.0. All datasets were processed individually but without dataset-specific hyperparameter optimization. The models were then evaluated on the corresponding test sets.

### Benchmarking of execution time

All methods were benchmarked on the same set of 512 × 512 px images after pre-loading dependencies to only measure the time to predict coordinates from an in-memory image. Time measurements were performed using Python’s datetime module on a MacBook Pro with a 2.4 GHz Quad-Core Intel Core i5 and 16GB 2133 MHz LPDDR3 Memory. Mean and standard deviation was measured on 100 images for seven rounds each.

### F1 integral score

The F1 integral score was selected as a metric to compare the detection and localization accuracy of given models. A linear distribution of 50 cutoff values was created between 0 and 3 px. At each cutoff value, ground truth and predicted coordinates with Euclidean distances below the given cutoff were matched using the Hungarian method (15). Finally, the trapezoidal rule was used to integrate the F1 score curve (Supplementary Figure S3). The integrated value was then normalized to the maximum area resulting in a minimum score of 0 and a maximal score of 1. An algorithmic overview is described in Supplementary Algorithm S1 of which an implementation can be found in deepBlink’s source code under deepblink.metrics.f1_integral.

### Root mean square error

The root mean square error (RMSE) was defined as the mean Euclidean distance of all true positive coordinates at a cutoff value of 3 px. True positive coordinates are predicted coordinates with an assignment to a ground truth coordinate.

(1) $$\begin{equation*} {\rm RMSE}(\hat{y}, y) = \sqrt{\sum ^n_{i=1}{\frac{(\hat{y}_i - y_i)^2}{n}}} \end{equation*}$$

## RESULTS AND DISCUSSION

**Figure 1.**
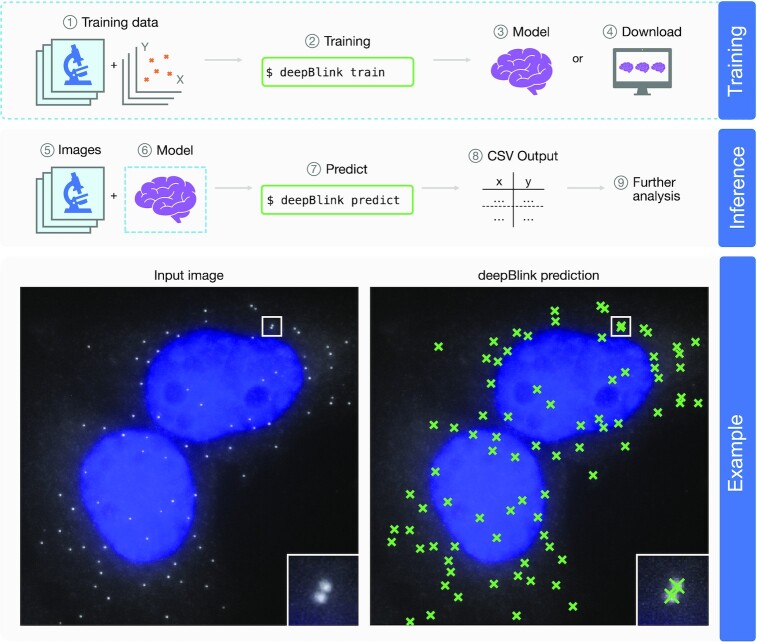
Overview of deepBlink’s functionality. deepBlink requires a pre-trained model that can be obtained by training from scratch using custom images and coordinate labels (1–3) or downloaded directly (4). To predict on new data, deepBlink takes in raw microscopy images (5) and the aforementioned pre-trained model (6) to predict (7) spot coordinates. The output is saved as a CSV file (8) which can easily be used in further analysis workflows (9). An example use case is shown for a smFISH analysis with blue indicating DAPI staining.

Here we present deepBlink, a novel neural network-based tool to detect and localize diffraction-limited spots. deepBlink can batch process images with different background brightness levels and spot qualities without relying on manual adjustments of thresholds. deepBlink’s interface provides the ability to detect and localize spots on images using a pre-trained model (Figure 1, steps 5–9). Pre-trained models are available for download using deepBlink (Figure 1, step 4). Alternatively, new models can also be trained in deepBlink using custom-labeled data (i.e. images and their corresponding spot-coordinate annotations) (Figure 1, steps 1–3). To customize training, the plug-and-play software architecture with a central configuration file allows more experienced deep learning practitioners to easily adjust existing functionalities or add new ones. Instructions on how to install and use deepBlink are described in a tutorial (https://youtu.be/vlXMg4k79LQ).

The current default network architecture is a fully convolutional neural network that exploits the U-Net architecture (13) to encode features that are used for spot coordinate prediction (Supplementary Figure S1). The network takes in microscopy data and maps the input images onto smaller regions we termed ‘grid-cells’. For each grid-cell, the network predicts the probability and localization of a spot within its region. This process works similarly to the region-based bounding-box predictions originally developed in the YOLO architecture (16).

Each grid-cell returns three values corresponding to the probability, the *x*, and the *y* position of a single spot. The performance of the network critically depends on the cell-size (width/height in pixels of one grid-cell in the original image) as too small sizes can lead to a class imbalance. Given a fixed number of spots in an image, decreasing the cell-size results in fewer grid-cells containing a spot. This will create an imbalance for the classification part of the loss, favoring background (grid-cells without spots). Images with higher spot densities perform better even at smaller cell-sizes (Supplementary Figure S2). In contrast, bigger cell-sizes increase the risk of one grid-cell containing multiple spots that pose the known design limitation as one grid-cell cannot regress to multiple coordinates (17). We found a cell-size of four to be ideal for most scenarios, as only around 0.0003% of grid-cells contained more than one spot across all datasets used (Supplementary Figure S2). Although a cell-size of four was found to be optimal, datasets containing significantly more or less dense regions of spots could require smaller or larger cell-sizes respectively. deepBlink allows for the configuration of cell-sizes when training new models.

The basic building blocks of our architecture consist of three convolutional layers followed by a squeeze and excitation layer (18). We employ spatial dropout layers (19) before every downsampling step to increase training stability and to reduce overfitting. We trained the models by minimizing the dice loss for the classification (*J*_class_, Equation 2) and the average root mean square error (RMSE) across all spots (*n*) for the localization (*J*_loc_, Equation 3). The objective function (*J*) is shown in Equation 4:

(2) $$\begin{equation*} J_{{\rm class}} = 1 - \frac{2 \left| \hat{y}_{{\rm class}} \cdot y_{{\rm class}} \right|}{\left| \hat{y}_{{\rm class}} \right| + \left| y_{{\rm class}} \right|} \end{equation*}$$

(3) $$\begin{equation*} J_{{\rm loc}} = \sqrt{\sum ^{n}_{i=1}{\frac{(\hat{y}_{{\rm loc},i} - y_{{\rm loc},i})^2}{n}}} \end{equation*}$$

(4) $$\begin{equation*} J = J_{{\rm class}} + 2 \cdot J_{{\rm loc}} \end{equation*}$$

To determine the importance of each component in our network, we performed ablation experiments where we removed individual elements to measure the decrease in performance (Supplementary Table S2). This showed us that the most important factor for model performance was the usage of dice loss. Dice loss has previously been shown to improve performance on class imbalanced datasets (20), which is common in spot detection as a majority of the image is background (i.e. not a spot). A greater weight on localization loss over detection loss also improved model performance. This is likely because it might be more challenging for the network to regress on spot coordinates rather than to detect spot-containing grid-cells. Another factor impacting performance was the use of the squeeze block. Squeeze blocks might allow for finer adjustments on the most important features by scaling convolutional feature maps (18). We experimented with other architectural blocks, namely inception (21) and residuals (22), that have been employed in similar computer vision tasks (23) but were not able to observe any significant improvements. We found that having a constant number of filters (64) across every layer of the network performs better than the typical increase/decrease in filters for every downsampling/upsampling step, respectively. This suggests that higher-level features found in deeper layers are less important for spot detection. We found 64 filters to be a good trade-off between model performance and model size. Furthermore, the reduction in overfitting by spatial dropout layers (19) was reflected in the lower performance upon ablation. Lastly, the bottom skip connection might allow easier gradient flow similar to residual blocks (22) if skipped layers are not useful or do not add value in overall accuracy.

To compare the performance of deepBlink to previously published methods, we used a new metric we termed the ‘F1 integral score’ (see Supplementary Figure S3 and Methods) that takes into account both spot detection and spot localization. The F1 integral score ranges between zero and one where one corresponds to perfect prediction and zero denotes that no prediction is within a 3px radius around the ground truth spot positions. Additionally, to compare detection efficiency and localization precision separately, we used the classical F1 score at a 3 px cutoff and the RMSE respectively. The F1 score measures the accuracy with which true positive spots are detected in a radius of 3 px. The F1 score ranges from zero to one where one denotes that all spots were detected and zero that no prediction is within a 3 px radius. To evaluate localization precision, the RMSE was calculated on true positive spots only. The RMSE is zero for perfect localization and increases as precision decreases.

To measure performance on a variety of spot sizes, shapes, densities and signal-to-noise ratios (SNRs), we used two experimental, manually labeled microscopy datasets (smFISH (11) and SunTag live cell single particle tracking (12)) and four synthetic datasets (a custom Particle dataset generated using (10) and the Microtubule, Vesicle, and Receptor datasets from the International Symposium on Biomedical Imaging (ISBI) particle tracking challenge (9)). Exemplary images of each dataset are displayed in Supplementary Figure S4. Since manual labeling is time-consuming, we measured how many images have to be labeled until only marginal performance increases are observed by adding more images. Based on the F1 integral score, a training set size of around 100 images is enough to achieve sufficient prediction quality on datasets with narrow SNR distributions (Supplementary Figure S5a, b), while more images are required for datasets with broader distributions (Supplementary Figure S5a, c). This is because broader SNR distributions have more variability and will thus require more labeled training examples for the networks to capture this variability.

We compared the performance of deepBlink against a popular classical method and a state-of-the-art deep learning-based method. TrackMate (3) is a Fiji plugin that uses the Laplacian of Gaussian (LoG) to detect and localize spots. This would require the setting of manual thresholds on an image-by-image basis for highly variable datasets. DetNet (8) is a more recent deep learning-based method that has been shown to perform well on the synthetic ISBI datasets. To improve the fairness of the comparison, we optimized hyperparameters for both benchmarking methods (TrackMate and DetNet) on every dataset individually (see Methods).

On average, deepBlink is able to significantly outperform other methods on all datasets (Table 1). We found that deepBlink performs excellently on all datasets both in detection (average efficiency above 85%, Supplementary Table S3) and in localization (average error below 0.5 pixels, Supplementary Table S4). TrackMate is able to slightly exceed our performance in detection efficiency on the Particle dataset and localization precision on the Receptor dataset. This is most likely because LoG filters are designed for particle-like objects. Importantly, however, deepBlink is able to outperform TrackMate on other particle-like datasets, specifically the experimental smFISH and SunTag datasets. Presumably, because experimental microscopy images have a higher image-to-image variability and thus our TrackMate thresholds that were determined based on the training set might not be optimal for the evaluated test set. In contrast, deepBlink outperforms DetNet across all datasets both in detection and localization. Taken together, while all of these methods can detect most spots, deepBlink still outperforms the other two pieces of software without any dataset-specific tuning. This will make deepBlink more accessible for a large variety of applications. Additionally, we fitted a Gaussian function on the original input data initialized by deepBlink’s prediction output and did not observe any improvement in localization (Supplementary Table S4). This further demonstrates deepBlink’s localization precision.

**Table 1.** F1 integral score (mean ± standard deviation) results for three methods across six datasets. Statistical significance was determined by one-sided Wilcoxon signed-rank test (deepBlink greater than) with **P < 1e^−2^, ****P < 1e^−4^. Size of test set: 129 smFISH, 105 SunTag, 64 Particle, 240 Microtubule, 240 Receptor and 240 Vesicle. Bold signifies the highest score

		TrackMate		DetNet		deepBlink
Real	smFISH	0.865 ± 0.177	****	0.442 ± 0.250	****	**0.905 ± 0.145**
	SunTag	0.652 ± 0.328	**	0.036 ± 0.040	****	**0.712 ± 0.279**
Synthetic	Particle	0.941 ± 0.008	****	0.828 ± 0.014	****	**0.944 ± 0.008**
	Microtubule	0.380 ± 0.228	****	0.298 ± 0.149	****	**0.637 ± 0.261**
	Receptor	0.606 ± 0.344	****	0.517 ± 0.258	****	**0.682 ± 0.310**
	Vesicle	0.459 ± 0.198	****	0.520 ± 0.245	****	**0.732 ± 0.259**
	Average	0.651 ± 0.214		0.440 ± 0.159		**0.769 ± 0.210**

We compared the execution time on a set of sample images between all methods and found that deepBlink is slower than both TrackMate and DetNet (TrackMate: 30 ± 6 ms, DetNet: 253 ± 31 ms, deepBlink: 502 ± 27 ms). Since TrackMate, however, requires manual adjustment of parameters on an image-by-image basis, the gain in execution speed is offset by the time required to manually set these parameters. DetNet is marginally faster than deepBlink, but as previously mentioned, has markedly worse performance.

To determine the effect of spot densities and SNRs on detection performance, we used the official categories from the ISBI particle tracking challenge and divided the Microtubule, Receptor, and Vesicle datasets accordingly (see Methods). In general, both spot density and SNR do not affect the performance of deepBlink except for very low SNRs where no model is able to perform well (Supplementary Table S5, Supplementary Figures S6 and S7).

deepBlink detects and localizes spots on 2D data, but importantly, one can easily use coordinate outputs for multi-dimensional data analysis. This allows deepBlink’s application in multi-channel colocalization, particle tracking across time, and three-dimensional spot detection. To this end, deepBlink is applied on every channel/frame/slice of a multi-dimensional image separately. Multi-dimensional detection is then achieved by linking identified particles. Example workflows are shown on GitHub (https://github.com/BBQuercus/deepBlink/tree/master/examples).

In summary, deepBlink enables automated detection and accurate localization of diffraction-limited spots in a threshold-independent manner. deepBlink is packaged in an easy and ready to use command-line interface and KNIME node, allowing both novel and expert users to efficiently analyze their microscopy data. All pre-trained models are available for download from Figshare (see Methods) or through the command-line interface. We benchmarked deepBlink on six publicly available datasets that contain both synthetic images as well as smFISH and live cell (SunTag) imaging data, each with a variety of spot sizes, shapes, densities, and signal-to-noise ratios. We show that deepBlink outperforms all benchmark methods, both in detection and localization across these datasets. We expect that our improved detection efficiency and localization precision will also enhance current particle tracking analyses and make image processing more reliable and reproducible.

## DATA AVAILABILITY

All datasets with their training, validation, and test splits are available on Figshare (https://figshare.com/projects/deepBlink/88118) together with all trained models used for benchmarking. Further data will be made available from the corresponding authors upon request.

deepBlink’s command-line interface is available on PyPI. Updated versions of the software can also be found on GitHub https://github.com/BBQuercus/deepBlink. The KNIME node is available on KNIME Hub (https://hub.knime.com/bbquercus/spaces/Public/latest/deepBlink).

## Supplementary Material

gkab546_Supplemental_File
