# Supplementary material for: deepBlink: threshold-independent detection and localization of diffraction-limited spots
Source: Nucleic Acids Res. 2021 Jul 1;49(13):7292–7. doi: 10.1093/nar/gkab546 (PMC8287908; doi:10.1093/nar/gkab546)
Supplement: gkab546_Supplemental_File [file gkab546_supplemental_file.pdf]

Supplementary Information

Algorithm 1: Algorithmic description of the F1 integral score

Data: Two lists of coordinates $\hat{y}$ (prediction), y (ground truth)

Result: F1 integral score

Function `hungarian_assignment(`V, W, *cost*`):`

l_w = `len(`W`)`

l_v = `len(`V`)`

L = `min(`l_w, l_v`)`

P_w = `permutations(`W`)`

P_v = `permutations(`V`)`

return (w, v) **for** $w \in P_w$, $v \in P_v$ such that $\sum_{i=0}^{L-1}$`cost(`w_i, v_i`)` is minimized

Function `assignment(`V, W, *cost*, *cutoff*`):`

(v, w) = `hungarian_assignment(`V, W, *cost*`)`

return $[(v_i,\ w_i)$ **for** $v_i,\ w_i$ in (v, w) **if** $\text{cost}(v_i, w_i) \leq \text{cutoff}]$

Function `calculate_f1_at_cutoff(`$\hat{y}, y$, *cost*, *cutoff*`):`

pred_true = `assignment(`$\hat{y}, y$, *cost*, *cutoff*`)` `/* assignment of` $\hat{y}$ `to` y `*/`

true_pred = `assignment(`$y, \hat{y}$, *cost*, *cutoff*`)` `/* assignment of` y `to` $\hat{y}$ `*/`

t_p = `len(`*true_pred*`)`

f_n = `len(`$\hat{y}$`)` - `len(`*true_pred*`)`

f_p = `len(`y`)` - `len(`*pred_true*`)`

$$\text{F1} = 2 * (P * R)/(P + R), \text{ where } \begin{cases} P = t_p/(t_p + f_p) \\ R = t_p/(t_p + f_n) \end{cases}$$

return F1

min_cutoff = 0

max_cutoff = 3

num_cutoff = 50

cutoffs = `linspace(`*min*=*min_cutoff*, *max*=*max_cutoff*, *num*=*num_cutoff*`)`

scores = [`calculate_f1_at_cutoff(`$\hat{y}, y$, *euclidean_dist*, *c*`)` **for** c in cutoffs]

$\text{F1}_{integral}$ = `trapezoidal_rule(`*scores*`)`

$\text{F1}_{integral}$ score /= max_cutoff `/* normalize between 0 and 1 */`

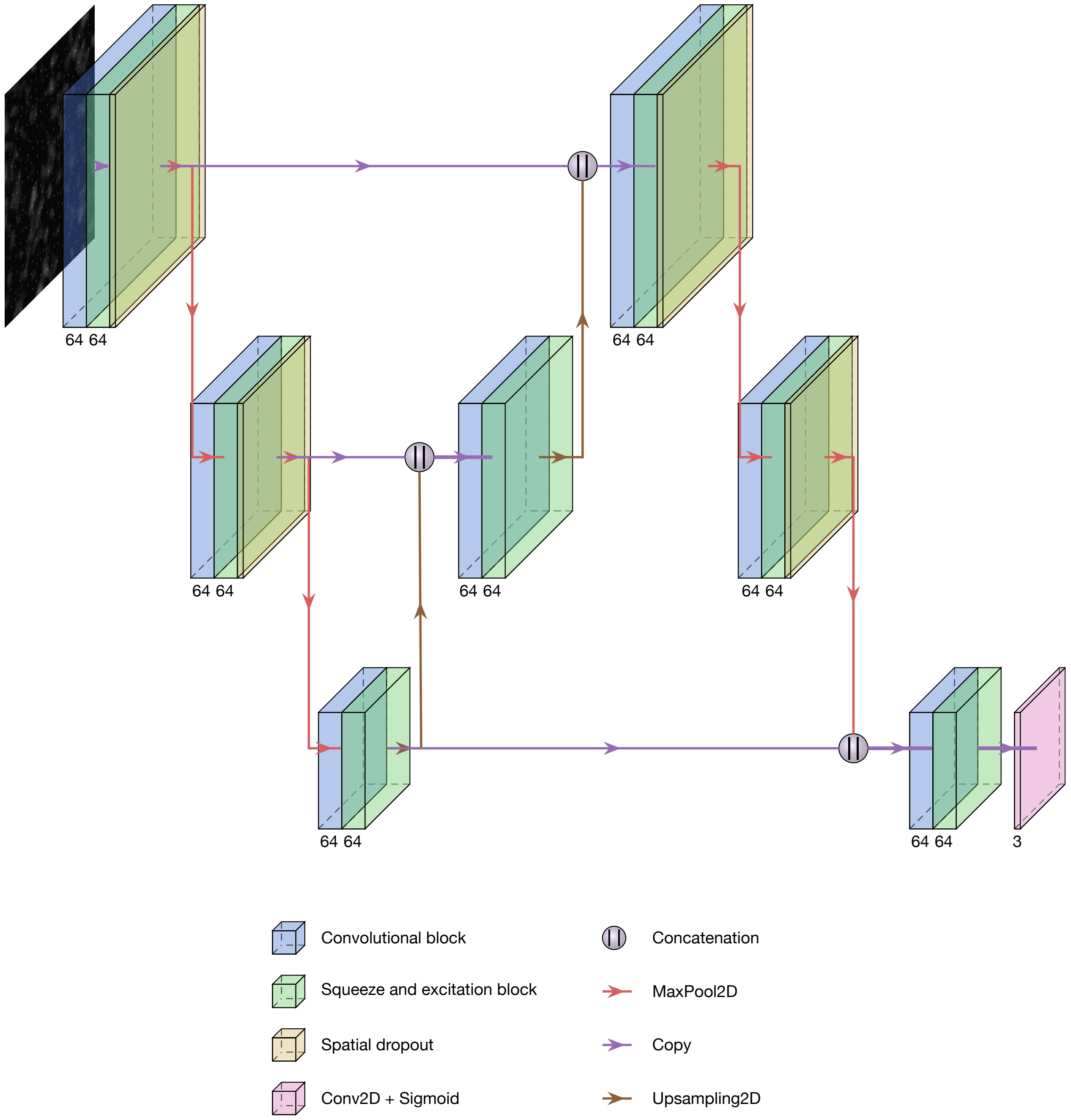

Supplementary Figure 1. Overview of the default neural architecture for deepBlink (components are described in Supplementary Table 1). The figure was created using modified PlotNeuralNet code (https://github.com/HarisIqbal88/PlotNeuralNet).

Supplementary Table 1. Description of network blocks in tabular format. Parameters that were not specifically listed were set to the default values of TensorFlow version 2.2.

(a) List of parameters in every "Conv2D" layer.

Parameter	Value
Kernel	3x3
Kernel initializer	He et al.
Padding	Same
Bias regularizer	L2 10^{-6}
Kernel regularizer	L2 10^{-6}

(b) Layers in the "Convolutional block".

Layer name	Operator	Input
conv2d_1	Conv2D	input
activation_1	LReLU	conv2d_1
conv2d_2	Conv2D	activation_1
activation_2	LReLU	conv2d_2
conv2d_3	Conv2D	activation_2
activation_3	LReLU	conv2d_3

(c) Layers in the "Squeeze and excitation block".

Layer name	Operator	Input
gap2d	GlobalAveragePooling2D	input
dense_1	Dense(units = max(n//8, 1))	gap2d
activation_1	LReLU	dense_1
dense_2	Dense(units = n)	activation_1
activation_2	Sigmoid	dense_2
multiply	Multiply	activation_2, input

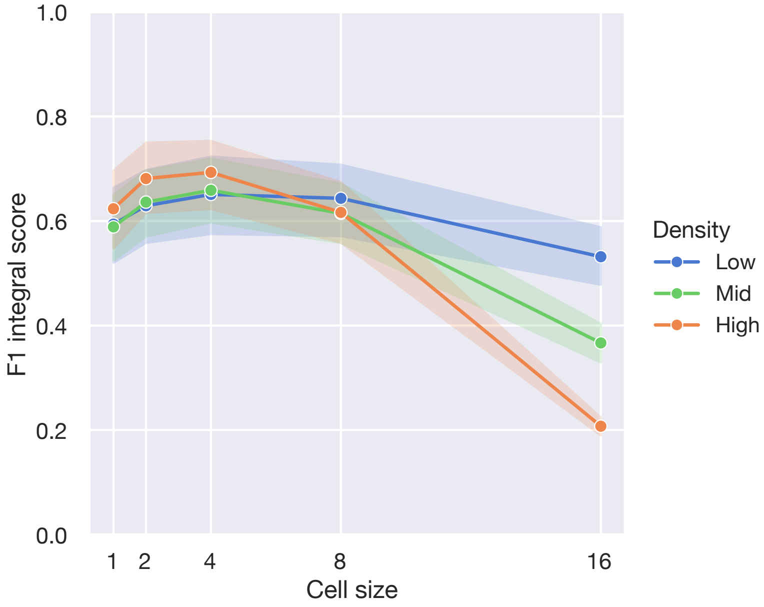

Supplementary Figure 2. Relationship between cell size and F1 integral score at low, mid and high spot densities for the Receptor dataset.

Supplementary Table 2. Ablation table on the smFISH and Receptor datasets showing F1 integral scores (mean ± standard deviation) and percentage decrease (%-dec) upon removal of singular features. Features were sorted in descending order of mean percentage decrease. Bold signifies the highest score. The "Replacement" column describes how each feature was replaced.

		smFISH		Receptor		Mean
	Replacement	Score	%-dec	Score	%-dec	%-dec
Full network		**0.905 ± 0.145**		**0.683 ± 0.309**		
Dice Loss	BCE	0.806 ± 0.196	10.990	0.639 ± 0.347	6.460	8.730
RMSE Weight	1/10	0.856 ± 0.129	5.450	0.675 ± 0.304	1.160	3.310
Squeeze Block	Remove	0.893 ± 0.140	1.300	0.656 ± 0.317	3.930	2.620
Filters 2^n	4	0.885 ± 0.145	2.200	0.673 ± 0.301	1.450	1.830
Spatial Dropout	Dropout	0.900 ± 0.143	0.610	0.666 ± 0.309	2.430	1.520
RMSE Weight	1/2	0.890 ± 0.135	1.670	0.678 ± 0.307	0.700	1.190
Filters 2^n	5	0.890 ± 0.144	1.610	0.680 ± 0.309	0.450	1.030
Bottom Skip Connection	Remove	0.895 ± 0.170	1.120	0.680 ± 0.308	0.370	0.750
Batch Size	1	0.900 ± 0.148	0.570	0.677 ± 0.309	0.850	0.710
Filter Arrangement	Symmetrical	0.897 ± 0.141	0.850	0.679 ± 0.309	0.560	0.710
Convolutional Blocks	Residual	0.903 ± 0.147	0.210	0.675 ± 0.311	1.080	0.650
Optimizer	ADAM	0.894 ± 0.154	1.190	0.682 ± 0.312	0.060	0.630
L2 Regularization	Remove	0.896 ± 0.165	1.020	0.682 ± 0.310	0.060	0.540
Conv2D Activation	ReLu	0.897 ± 0.153	0.900	0.683 ± 0.312	-0.010	0.450
Convolutional Blocks	Inception	0.898 ± 0.137	0.740	0.683 ± 0.309	0.000	0.370
Filters 2^n	7	0.903 ± 0.146	0.200	0.680 ± 0.312	0.400	0.300
Batch Size	4	0.902 ± 0.150	0.330	0.682 ± 0.309	0.090	0.210

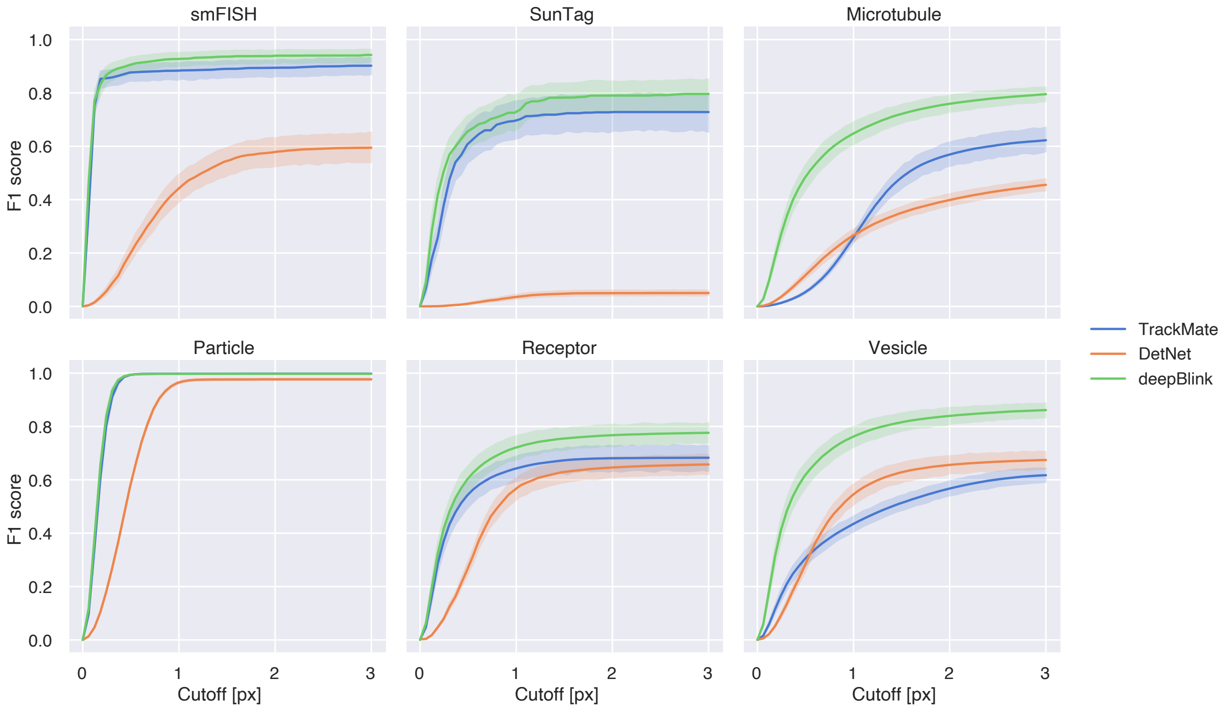

Supplementary Figure 3. F1 score as a function of cutoffs on all datasets across and methods. Shaded areas correspond to the confidence interval of 95%.

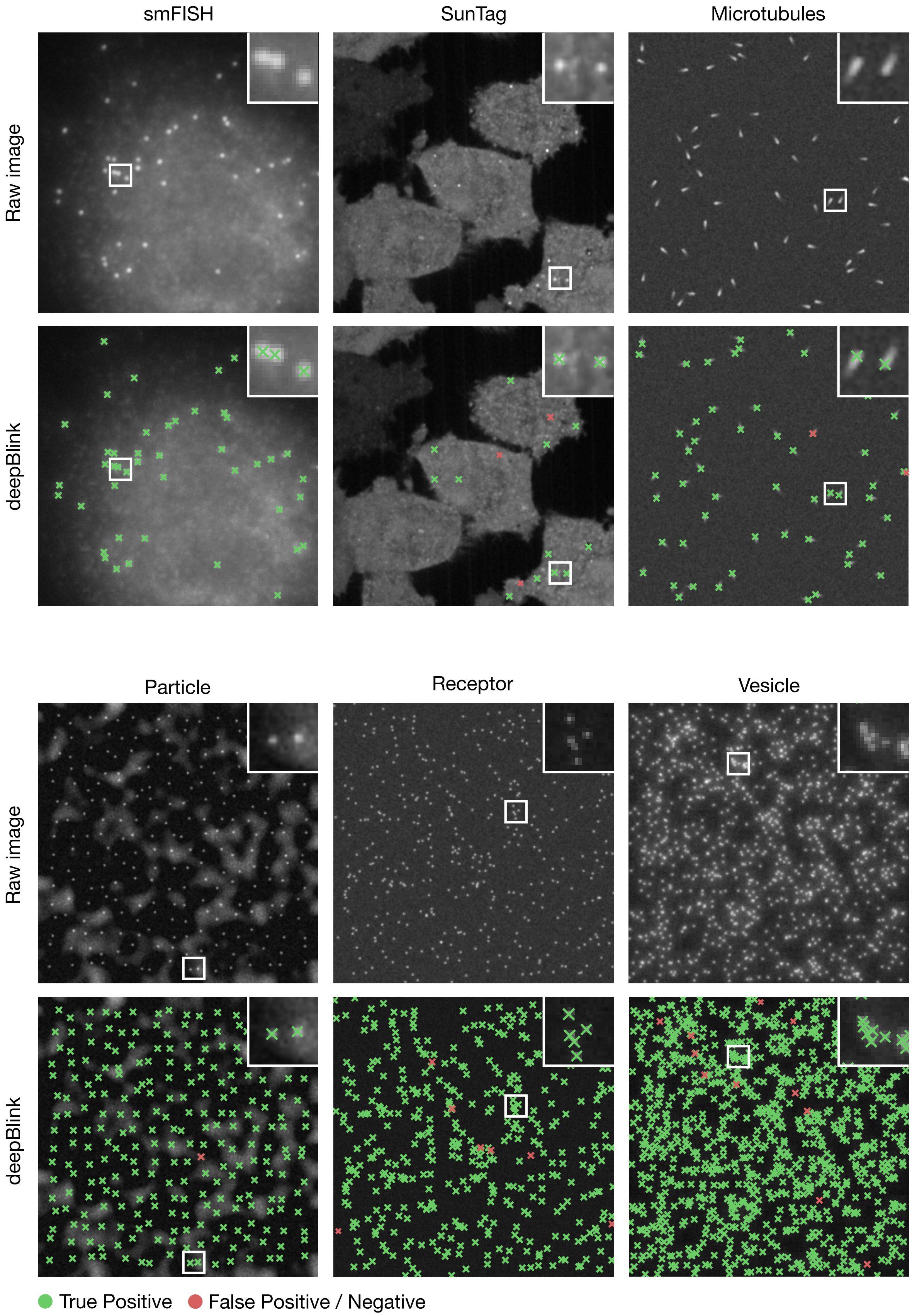

Supplementary Figure 4. Representative images for all six datasets with their corresponding prediction.

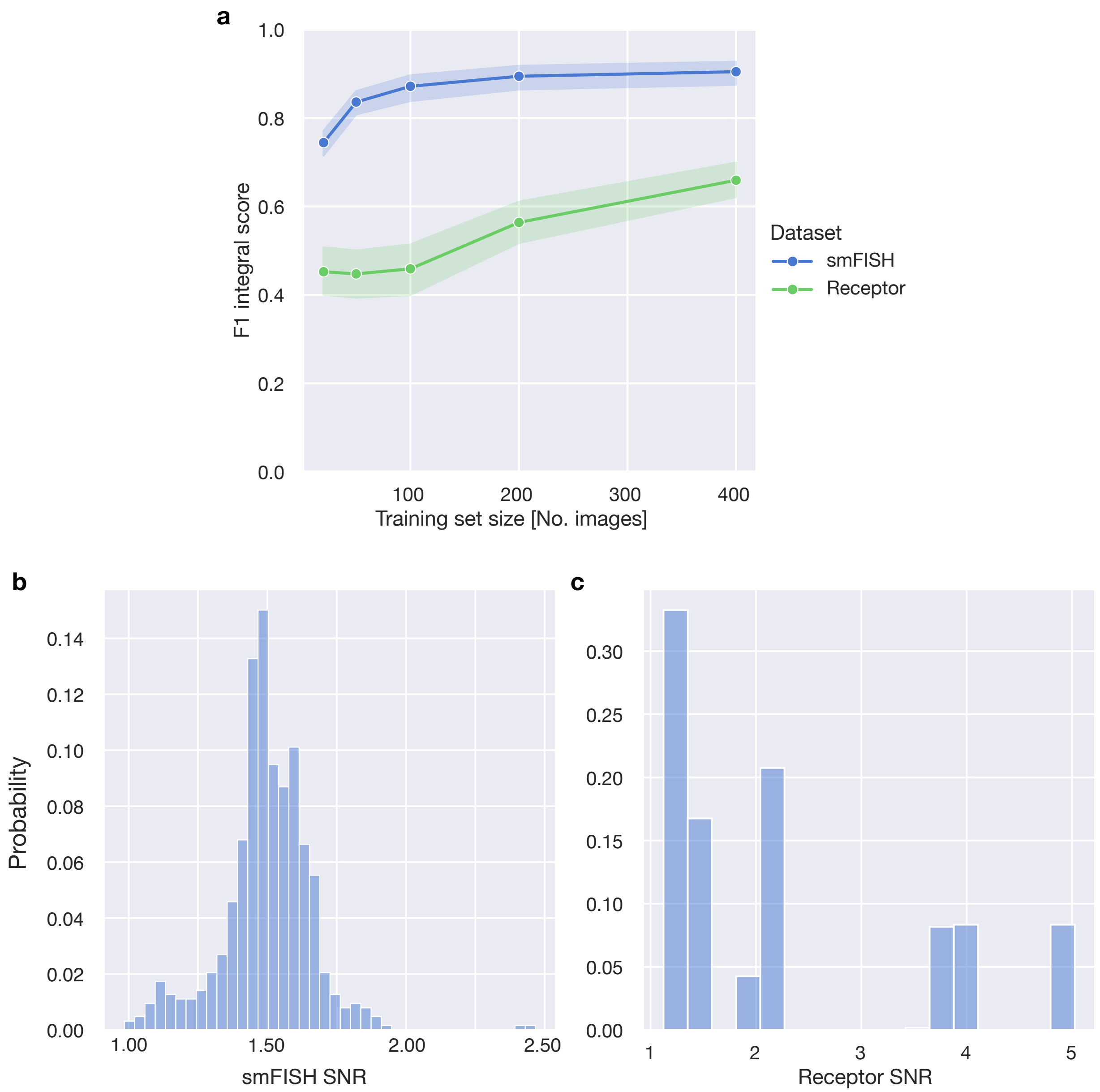

Supplementary Figure 5. Effect of dataset size on model performance. **a** Relationship between training set size and F1 integral score on the same holdout test set. Shown for the smFISH and Receptor datasets. **b**, **c** show the SNR distribution across the entire dataset for smFISH and Receptor respectively.

Supplementary Table 3. F1 scores at 3px cutoff of deepBlink and benchmarking methods (mean ± standard deviation). Shows values for three methods across six datasets. Higher value is better. Bold signifies the highest score.

		TrackMate	DetNet	deepBlink
Real	smFISH	0.902 ± 0.183	0.595 ± 0.319	**0.942 ± 0.141**
	SunTag	0.728 ± 0.362	0.050 ± 0.053	**0.796 ± 0.297**
Synthetic	Particle	**0.998 ± 0.002**	0.977 ± 0.014	0.997 ± 0.002
	Microtubule	0.623 ± 0.366	0.456 ± 0.169	**0.795 ± 0.214**
	Receptor	0.683 ± 0.367	0.658 ± 0.288	**0.776 ± 0.300**
	Vesicle	0.617 ± 0.216	0.674 ± 0.258	**0.861 ± 0.213**
	Average	0.759 ± 0.250	0.568 ± 0.183	**0.861 ± 0.195**

Supplementary Table 4. RMSE values of deepBlink and benchmarking methods (mean ± standard deviation). RMSE was only calculated on true positive spots. Shows values for three methods across four synthetic datasets, where the exact spot position is known. Lower value is better. Bold signifies the lowest score.

	TrackMate	DetNet	deepBlink + Gaussian	deepBlink
Particle	0.167 ± 0.020	0.438 ± 0.016	0.207 ± 0.021	**0.157 ± 0.020**
Microtubule	1.017 ± 0.221	0.887 ± 0.228	0.850 ± 0.223	**0.588 ± 0.339**
Receptor	**0.428 ± 0.255**	0.661 ± 0.203	0.517 ± 0.302	0.435 ± 0.294
Vesicle	0.658 ± 0.324	0.684 ± 0.223	0.644 ± 0.312	**0.459 ± 0.311**
Average	0.568 ± 0.205	0.667 ± 0.167	0.554 ± 0.215	**0.410 ± 0.241**

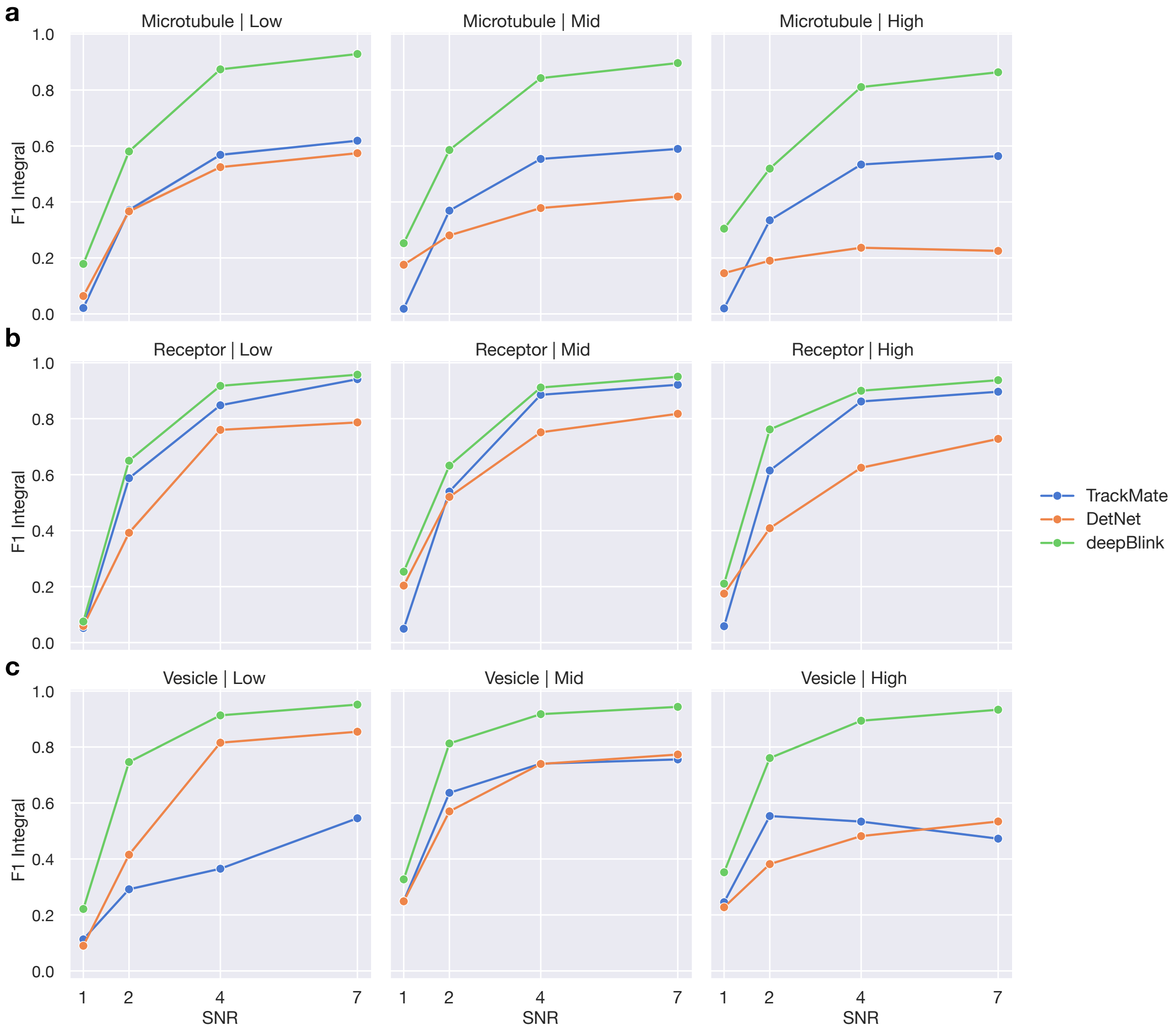

Supplementary Figure 6. Mean F1 integral scores of all three methods at different spot densities and image SNRs for the **a** Microtubule, **b** Receptor, and **c** Vesicle datasets.

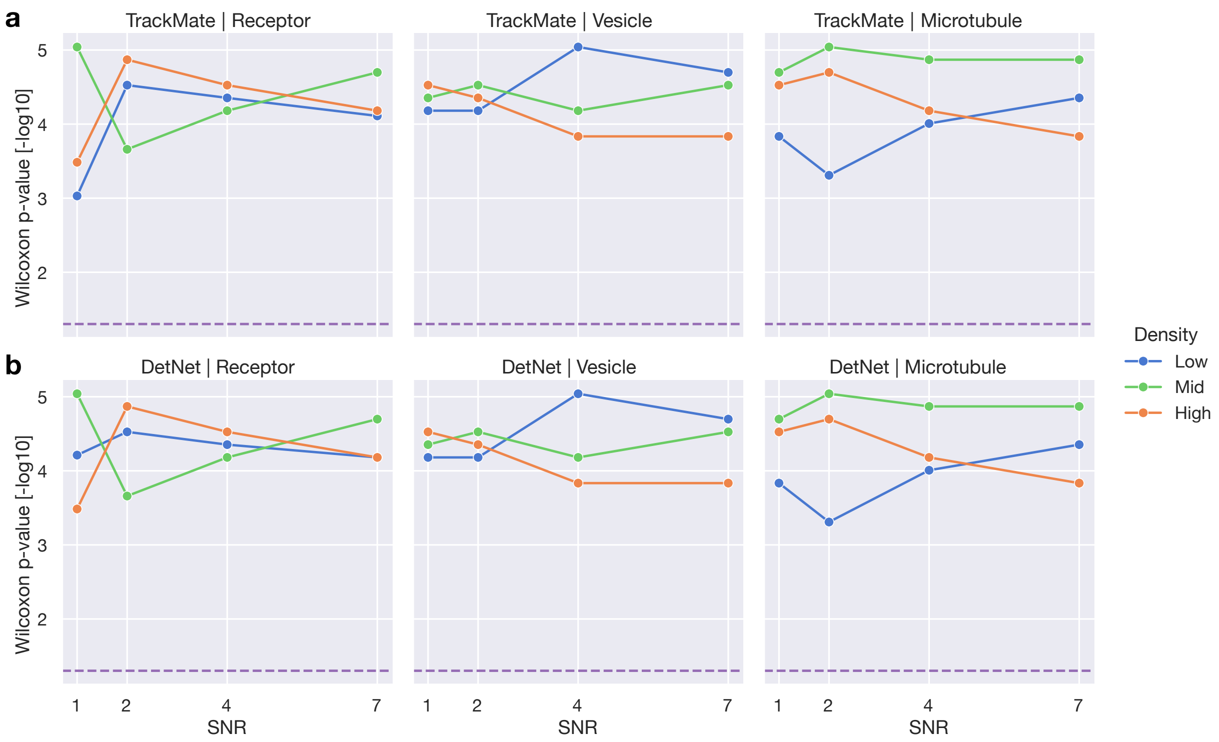

Supplementary Figure 7. P-Values from one-sided Wilcoxon signed-rank tests measuring how significantly deepBlink performs better than **a** TrackMate and **b** DetNet at different spot densities and image SNRs. The purple dotted line indicates a significant p-value of 0.05.

Supplementary Table 5. F1 integral score (mean ± standard deviation) for images of SNR equal to one. Shows values for three methods across three datasets.

Dataset	Density	TrackMate	DetNet	deepBlink
Microtubule	Low	0.021 ± 0.017	0.064 ± 0.014	**0.179 ± 0.022**
	Mid	0.018 ± 0.008	0.176 ± 0.015	**0.253 ± 0.012**
	High	0.020 ± 0.006	0.145 ± 0.012	**0.305 ± 0.007**
Receptor	Low	0.052 ± 0.029	0.059 ± 0.013	**0.075 ± 0.013**
	Mid	0.050 ± 0.010	0.204 ± 0.013	**0.253 ± 0.013**
	High	0.059 ± 0.010	0.175 ± 0.009	**0.211 ± 0.010**
Vesicle	Low	0.113 ± 0.016	0.090 ± 0.011	**0.221 ± 0.031**
	Mid	0.250 ± 0.016	0.249 ± 0.010	**0.327 ± 0.013**
	High	0.246 ± 0.008	0.228 ± 0.008	**0.353 ± 0.008**

Supplementary Table 6. The set of optimized parameters used for each benchmarking method and for each dataset.

(a) TrackMate

Dataset	Diameter	Quantile
smFISH	4.0	0.871
SunTag	1.0	0.170
Particle	4.0	0.001
Microtubule	4.0	1.091
Receptor	3.0	0.795
Vesicle	3.0	1.342

(b) DetNet

Dataset	Alpha
smFISH	0.4737
SunTag	0.6842
Particle	0.5263
Microtubule	0.2105
Receptor	0.8946
Vesicle	0.5789
